# Popular Fallacies

**Published:** 1855-04

**Authors:** 


### HALL'S JOURNAL OF HEALTH.

VOL. II.] APRIL, 1855. [NO. IV

### POPULAR FALLACIES.

It is a great mistake, that a morning walk or other form of exercise before breakfast is healthful; the malaria which rests on the earth about sunrise in summer, when taken into the lungs and stomach, which are equally debilitated with other portions of the body from the long fast since supper, is very readily absorbed and enters the circulation within an hour or two, poisoning the blood, and laying the foundation for troublesome diseases; while in winter the same debilitated condition of these vital organs readily allows the blood to be chilled, and thus renders the system susceptible of taking cold, with all its varied and too often disastrous results.

I do not wish to dismiss the statement which I have made with a simple assertion. The denial of what is almost universally considered a truth so palpable, as scarcely to admit of proof, may well challenge investigation. Besides, I do not want the regular readers of the Journal to have their memories crowded with abstract precepts and pithy saws about health; I desire them, on the contrary, to become masters of general principles, to know and to understand the reason of things; then, these things can be remembered without an effort, while the principle being known, a very varied application is easily made and practically observed, a striking example of which is given in the March number, in reference to the prompt cure of poisons and bites and stings of insects and reptiles by the employment of familiar articles of kitchen use.

What I shall say on the subject of morning exercise is intended to apply mainly to all sedentary persons, those whose employment is chiefly indoors. And here I will simply appeal to the actual experience of any sedentary reader if he has not before now noticed, when he has been induced from some extraordinary reason to take active exercise before breakfast on some bright summer morning, that he felt rather a less relish for his food than usual; in fact had no appetite at all; there was a certain sickishness of feeling, with a sensation of debility by no means agreeable. It will be said here, this was because it was unusual, that if followed up these feelings would gradually disappear. If that is so, it is but a negative proof, for the system naturally has an inherent resisting power called into action by hurtful appliances. A teaspoon of brandy will produce slight symptoms of lightness of head in some persons if taken before breakfast, but if continued, the same amount will, after a while, produce no appreciable discomfort; the cases are precisely parallel; that a man gets used to drinking brandy is no proof that it does not injure him.

Another person will remind me that the early air of a summer's morning seems so balmy and refreshing, so cool and delightful, that it cannot be otherwise than healthful. That is begging the question; it is a statement known by scientific observers to be not simply untrue, but to be absolutely false. It is a common observation in New Orleans, where I lived a number of years, by those who remain in the city during the raging of yellow fever, that when the air of mornings and evenings appears to be unusually delicious, so clear and cool and refreshing, it is a forerunner of an increase of the epidemic. Like the deceitful Syren, it destroys while it lures.

The fruitful cause of fevers and other epidemics in southern climes is the decomposition of vegetable matter: the ranker and more dense the vegetation, the more deadly are the diseases of that locality; this decomposition cannot take place without moisture and heat approaching ninety degrees of Fahrenheit. We are all familiar with the sad fact, that thousands upon thousands who have endured the hardships of mining in California have taken the "*Isthmus fever*" on their return, and lingered and died. From the first discovery of gold in the Sacramento valley the newspaper press was united in its cautions against the almost certain death attendant on sleeping at Chagres a single night, and even now it is considered one of the most important effects of the railroad *finished across the isthmus*, that passengers do not land at all at Aspinwall, but get into the cars at once and cross to Panama, where a *steamer is always in waiting* to receive passengers for San

Francisco, thus avoiding a night on the isthmus. Before the removal of the landing from Chagres to Aspinwall, it became common to make arrangements to remain on board the steamers until the passengers were ready to start immediately for Panama. All these precautions forced themselves on public attention. Now why was all this? Simply to avoid breathing the concentrated malaria arising from such immeasurable quantities of decaying vegetation shooting out of swamps and stagnant marshes, and so dense as to make penetration by man or beast impracticable.

The night was more dreaded than the day, for the following reason: The great heat of the sun caused a rapid evaporation of the malaria, rarifying it to such a degree that it almost instantaneously ascended to the upper atmosphere after the first morning hours; but in the course of the day, when the sun declines in power, these vapors gradually condense, get heavier, and fall to the earth, thus giving the layer of air within fifteen feet of the surface, a density and concentration of malaria malignantly fatal; while in the morning this density is not diminished until the sun has gained some power.

The older citizens of Charleston will tell you, that in early years, it was certain death for a stranger to sleep in the city one night, that during the most violent ragings of epidemics, citizens themselves, would not go to town to attend to necessary business, except at noon-day, the hottest portion of the twenty-four hours, because, then the malaria was most rarified and found by observation to be least hurtful. Few knew the reason, but the fact was so palpable, that its propriety enforced practical attention.

In the old books which treat of the terrible plagues which depopulated the large cities in the middle and earlier ages, the people who could not leave town, retreated to the upper stories of their dwellings, and would not come down to purchase necessary marketing from the country people, but would let down baskets by ropes, and draw up their provisions, and thus escaped with impunity, to a considerable extent; these were the practical results which followed the observation of actual facts, by a comparatively rude and unthinking age, and we unfortunates of the nineteenth century, who cannot leave the city in summer, but must have our noses always at the grindstone, whose mills stop when absent for a single day; we doctors who never have a leisure day or night, or hour, who always have a greater or less number who are looking up to us for life; looking to the hour of our anticipated visit as the happiest of the whole twenty-four; and we poorer Editors, who could not go if we would, otherwise our children would go supperless to bed: I say, we all may gather a practical lesson of great value from the customs of those of a far ruder age, a lesson which if learned well, and acted on, would save to us many a darling child, many a life's only hope, many a poor heart's only comfort—thus

Never allow your children to leave the second or third story in the morning until they have had a plain hearty breakfast; and send them up stairs within half an hour after sun down, or give them their supper at sundown: these observances ought to be adhered to from May until October in the North, and from April to November in the South. A rigid attention to this, would prevent at once, half the diarrhœas and summer complaints, and croups which desolate our hearths and hearts so often in summer time in the city.

It is a striking argument for the perversity of human nature, and one which often forces itself upon the attention of observant men, that we bolt a concentrated untruth without wincing, while what is true, with all its simplicity and beauty, and usefulness, is disputed inch by inch, with a suspiciousness and a pertinacity most remarkable.

So it will be, I have no doubt, with the sentiment I have advanced; instead of being received, and acted upon, many a mind will be busied in finding an argument against it, instead of considering the force of the proof offered for it, just as we all have many times observed when ordinary minds are engaged in an argument, it will occur in perhaps nine cases out of ten, that the listener's whole attention is occupied in casting about for an objection or new proof, instead of weighing the argument of the speaker; consequently, at the end of the dispute, neither party is a whit the wiser, but rather more confirmed in his previous opinion, from the fact that no argument or proof to the contrary was allowed a hearing. I will just step aside a moment here to make a useful suggestion, for being "*free born*," and in a remarkably "*free country*," so said at least; so free indeed, that if you differ from any body else upon any subject, or fail to walk in the exact track of your predecessors, or do or say any thing different from Mr. Everybody, you are considered a ninny, or a mule; being as I just said, a citizen of this remarkably free and tolerant country, why should I be bound to stick to the literal text for six or eight pages; persons meandering along the cow paths in the woods, like to step aside occasionally and pick an inviting flower, which otherwise would have wasted its sweetness on snakes, lizards and spiders; so I step aside from the consideration of disease and malaria, and cull a flower for my reader, relative to argumentation. It is such an important truth, so easily practiced, would save so many hard words, and harder thoughts, so many wounded feelings, so much love's labor lost, and by the way accomplish so much good, that really I think it is worth the whole year's subscription price to the Journal—it is this:

*If you want to convince anybody of anything, argue alone.*

Having delivered ourselves of this great and useful apothegm, we will resume the thread of the argument, taking it for granted, that the reader has not forgotten the subject matter of discussion, it being so imaginatively delightful—*a summer morning's walk.* It sounds charmingly, it brings with its mere mention, recollections so mournfully pleasing, or associations so delightful, that we long for the realization, at least until "sun up" to-morrow, then what a change! we would not give one half awake good stretch, one five minutes' second nap, for all the summer morning walks of a whole year. Who does not feel that the *vis inertia* of the first waking moments of a May morning, is worth more than a dozen rambles before breakfast. I am for the largest liberty of enjoyment; I am not among the multitude of the weak minded folk, the negative sort of minds, to discard what is good to eat or drink, or enjoy, for no other reason, that I can perceive, than that it is good, and a cross is meritorious. One man says tea is injurious; another Solomon avers that coffee makes people bilious, a third, and he a Broadway author too, has written a whole book to prove that if we eat wheat bread, it will make our bones brittle, and that if we live to get old at all, the first time we fall, we'll break all to pieces like a clay pipe-stem. Verily this is a free country, for if everybody is to be believed, we are free to eat nothing at all. So I do not advise a denial of that most deliciously enjoyable entity, a summer morning's nap, because it is for the reasons I have named, more healthful than the so lauded "*exercise before breakfast;*" if you must remain in bed until breakfast, or be out in the open air an hour or two before breakfast, on an empty stomach, then I say, as far as health is concerned, the nap is better than the exercise, for the incontrovertible reasons I have already given.

It requires no argument to prove the impurity of a city atmosphere about sunrise and sunset, reeking as it must, with the odors of thousands of kitchens and cesspools, to say nothing of the innumerable piles of garbage which the improvident poor allow to accumulate in front of their dwellings, in their back yards and their cellars; any citizen may satisfy himself as to the existence of noisome fumes by a summer evening's walk along any of our by-streets; and although the air is cooler in the mornings, yet the more hurtful of these malaria saturate it, but of such a subtle nature are they, that no microscopic observation, no chemical analysis has as yet been able to detect, in an atmosphere thus impregnated, any substance or subsistence to which these deadly influences might be traced, so subtle is the poison, so impalpable its nature; but invisible, untraceable as it may be, its influence is certain and immediate, its effects deadly.

Some will say, look how healthy the farmer's boy is, and the daily laborers, who go to their work from one year's end to another by "crack of dawn!" My reply is, if they are healthy, they are so in spite of these exposures; their simple fare, their regular lives, and their out-door industry, give their bodies a tone, a vigor, a capability of resisting disease, which nullifies the action of malaria to a very considerable extent. Besides, women live as long as men, and it cannot be said that they generally exercise out of doors before breakfast.

Our Knickerbocker ancestry! the very mention of them suggests—fat! a double fatness in fact—fat as to body and fat as to purse; if you catch hold of one of them, instead of getting a little pinch of thin skin, as you would from a lean Yankee, you clutch whole rolls of fat, solid fat—what substantial people the real, identical, original old Knicks are! how long they live too! expectant sons-in-law echo, sighingly, "*how long!*" in fact, I do not recollect of their dying at all, at least as we do; they simply ooze out, or sleep away. May we not inquire if there is not at least some connection between their health as a class, and the very general habit of the sons here, derived from their sires in fatherland, of eating breakfast by candle-light? Another very significant fact in point is, that the French in the south are longer lived, and suffer far less from the fevers of the country than their American neighbors; in truth, their exemption is proverbial; and as a class they have their coffee and boiled milk, half and half, with sugar, brought to their bedsides every morning, or take it before they leave the house.

It is not an uncommon thing for persons to go west to select a new home for their rising families, never to return: "*took sick and died;*" this is the sad and comprehensive statement of the widowed and the fatherless, owing doubtless, in many instances, to their travelling on horseback early in the morning and late in the evening in order to avoid the heat of the day.

*Many a traveller will save his life by taking a warm and hearty breakfast before starting in the morning, and by putting up for the night not later than sundown.*

It is of considerable practical importance to answer the question, why more persons have died in "the States" from Isthmus fever than in California? Simply, because on their way out, their bodies are comparatively vigorous, and there is in addition a degree of mental and moral excitement, which repels disease; but on the return, it is strikingly different; the body is wasted by hardship and privation, while the spirit is broken by disappointment, or the mind falls into a species of exhaustion, when successful, from the long and anxious strife for gold: both causes operating, one to weaken the body, the other to take away all mental elasticity; it is no wonder that the whole man becomes an easy prey to disease.

In subsequent numbers I may discuss other "POPULAR FALLACIES" in reference to the all-important subject of health. A whole number could be easily filled with them; but it was not my intention to tell too much at once, it would not be remembered; and then again, Wifey has several times given a gentle but a very decided admonition, "*Thy Journal reads very well, William, but I am afraid thee will give out.*" I have, however, a ready quietus to these groundless apprehensions, in a basket under my table, well filled with scraps, each of which affords matter for a leading editorial. The truth is, when I think it all over, the world has so many things to learn and unlearn, I am afraid I will get gray—what a delightful Tense that is—before I can set it right at all points, my ideas of right, and propriety, and truth, being considered the standard! What a vain creature is poor know-nothing man! how little indeed does the wisest of us rightly and truly know!

